# Enhancing climate resilience: A data-driven north rift weather prediction system for real-time forecasting and agricultural decision support

**DOI:** 10.1016/j.heliyon.2025.e42549

**Published:** 2025-02-07

**Authors:** John W. Makokha, Peter W. Barasa, Geoffrey W. Khamala

**Affiliations:** a Department of Science, Technology and Engineering, Kibabii University, Bungoma, Kenya; b Department of Computer Science, Kibabii University, Bungoma, Kenya

**Keywords:** North rift, Climate resilience, Data-driven, Weather prediction, Real-time forecasting and agricultural decision support, North rift weather prediction system

## Abstract

This study presents the development and integration of predictive models for the Normalized Difference Vegetation Index (NDVI) and Bare Soil Index (BSI) using the XGBoost algorithm within the North Rift Weather Prediction System (NRWPS) to enhance ecosystem monitoring in Kenya's North Rift region. Trained on a comprehensive dataset spanning 1995 to 2020, which includes precipitation (from the Climate Hazards Group InfraRed Precipitation with Station data (CHIRPS)), temperature (TerraClimate), historical NDVI (Landsat 4–5 Thematic Mapper (from 1995 to 2013) and Landsat 7 Enhanced Thematic Mapper plus (ETM+) (from 2014 to 2020)), and BSI (SoilGrids) data, the models effectively capture the complex relationships between environmental factors and vegetation health. The BSI model achieved an MSE of 0.029, an MAE of 0.019, and an R-squared score of 0.93, while the NDVI model yielded an MSE of 0.002, an MAE of 0.024, and an R-squared score of 0.945. These results demonstrate the models' strong predictive accuracy, enabling precise assessments of vegetation health and bare soil exposure. By analyzing temporal variations in vegetation health and land degradation from 1995 to 2020, the study identifies a significant inverse relationship between NDVI and BSI, where increasing bare soil exposure corresponds to declining vegetation health. The analysis also reveals that climatic factors particularly temperature (minimum and maximum) and precipitation play a critical role in shaping these trends, with high temperatures after 2000 associated with reduced NDVI, while regions with higher precipitation show healthier vegetation and lower BSI. The successful development of the NRWPS model provides significant opportunities for informing land management strategies, conservation efforts, and agricultural practices, enabling data-driven decision-making. Moreover, its integration into larger decision support systems allows for proactive interventions to mitigate land degradation and climate change stressors. This study emphasizes the importance of sustainable land-use practices and climate adaptation strategies to preserve vegetation health and manage ecosystem vulnerabilities effectively in the wake of regional climate change with the North Rift region most affected.

## Highlights

• XGBoost Boosts NRWPS: NDVI and BSI integration improves predictive accuracy.
• Comprehensive Monitoring: NRWPS integrates environmental data for land management.
• Climate Impacts Vegetation: Post-2000 temperature rise causes loss; rainfall aids recovery.
• Land Degradation Effect: Rising BSI, declining NDVI indicate soil exposure, vegetation loss.
• Sustainability & Resilience: NRWPS supports adaptation, food security, and conservation.

## 1 Introduction

**Fig. 1:**
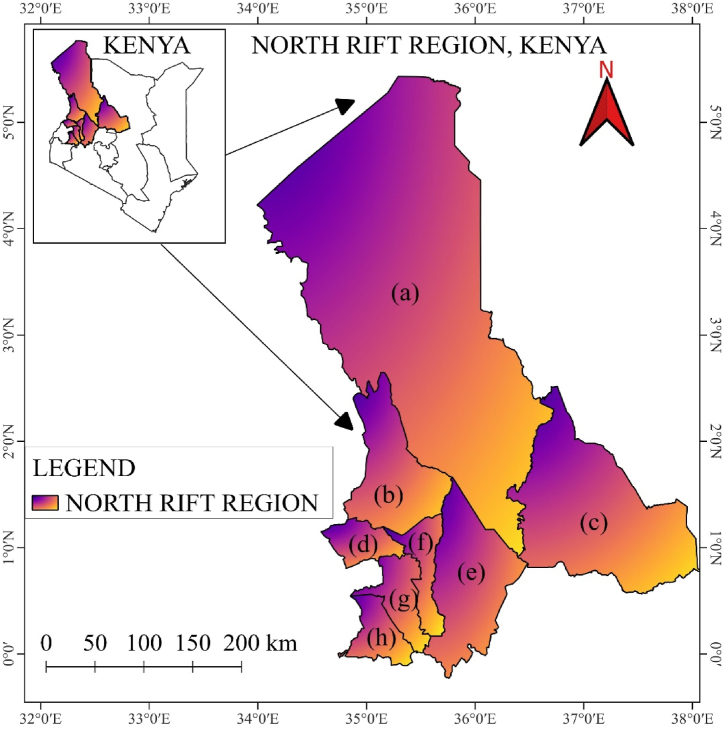
Map of North Rift Region, Kenya, highlighting the counties of (a) Turkana, (b) West Pokot, (c) Samburu (d) Trans Nzoia, (e) Baringo, (f) Elgeyo-Marakwet, (g) Uasin Gishu, and (h) Nandi.

The North Rift region of Kenya comprises eight counties in the North Rift region: TransNzoia, UasinGishu, Nandi, Turkana, Baringo, West Pokot, Samburu, and Elgeyo Marakwet as shown in Fig. 1. These counties heavily rely on rain-fed agriculture, making effective weather forecasting and environmental monitoring essential for boosting productivity and ensuring sustainable land use [1]. However, traditional weather prediction systems often provide limited insight into localized environmental conditions, which is crucial for informed agricultural decision-making [2]. In an attempt to address this challenge, the North Rift Weather Prediction System (NRWPS) incorporates advanced environmental indices alongside weather data to provide a more comprehensive monitoring tool for accelerated and real-time regional forecasting and agricultural decision-making to support farmers and land managers has been proposed.

To develop a robust predictive model such as NRWPS, there is a need to understand the intricate relationships between vegetation dynamics, hydrological processes, and climatic variables [3]. This understanding effectively strengthens sustainable management strategies in climate-sensitive regions such as the North Rift region of Kenya. Before then, there is a need to understand the coherency and phase delays between the Normalized Difference Vegetation Index (NDVI), precipitation, and temperature among other climatic variables either regionally or globally. Globally, studies have demonstrated the coherency and phase delay between NDVI, water flow, precipitation, and temperature have been explored across diverse climatic and ecological settings [3,4]. In temperate grasslands of China, studies revealed that increased summer precipitation and autumn temperatures significantly delayed the end of the growing season, with variations influenced by nighttime and daytime temperature changes across different vegetation types [3,4]. These findings emphasize the need for considering regional climatic factors in modeling vegetation phenology. Similarly, in northern Canada, long-term analyses of river flow trends in basins like the Athabasca River Basin demonstrated the influence of rising temperatures on winter water flow through snowpack melt, alongside precipitation-induced variations in summer flow [5].

Additionally, research on extreme drought events in southeastern Australia showcased the sensitivity of NDVI to combined meteorological stressors, such as a lack of precipitation and elevated temperatures, underlining the utility of vegetation indices in monitoring drought impacts [6]. Meanwhile, investigations into the relationship between surface temperature and NDVI in ecosystems like Brazil's Cananéia–Iguape Coastal System revealed significant correlations that varied seasonally and spatially, highlighting the nuanced ways vegetation responds to climatic factors [7,8]. Collectively, these studies underscore the critical role of climatic drivers in shaping vegetation dynamics, hydrological regimes, and ecosystem resilience while demonstrating the value of integrating NDVI with precipitation, temperature, and water flow data to enhance predictive models and inform sustainable management strategies. Building on these global insights, this study focuses on developing a data-driven NRWPS to enhance climate resilience and agricultural decision-making in the North Rift region of Kenya. By integrating NDVI, precipitation, and temperature, the NRWPS aims to provide high-resolution, real-time forecasts.

The NRWPS leverages key indices such as the NDVI and Bare Soil Index (BSI), which offer insights into vegetation health and soil exposure. These indices, combined with machine learning techniques like XGBoost, allow the system to deliver high-accuracy predictions. By providing detailed, real-time information about land conditions, the system enables users to plan more effectively for crop management, irrigation scheduling, and land conservation. This integration of environmental and weather data helps reduce risks associated with climate variability [9]. Furthermore, incorporating NDVI and BSI predictions into the NRWPS equips agricultural stakeholders with critical data that supports sustainable land management and enhances agricultural productivity.

This holistic approach also aligns with broader efforts to strengthen food security in the region, offering a comprehensive tool for both current and long-term planning. By providing detailed information on both weather and land conditions, the system allows for more adaptive and resilient agricultural practices. The success of the NRWPS highlights its potential for further development and scaling. By engaging local communities and continuously refining the system's predictive capabilities, NRWPS can have a lasting impact on regional agriculture and environmental management. As the system evolves, it will be an invaluable resource for promoting climate resilience and sustainable land use in the North Rift region, contributing to broader goals of food security and adaptation to climate change.

As stated earlier, the North Rift region of Kenya predominantly relies on rain-fed agriculture which is under threat due to escalating climate variability, with unpredictable weather patterns threatening crop yields, livelihoods, and food security. Local communities predominantly small-scale farmers often lack timely and accurate weather information, leaving them susceptible to risks such as droughts, floods, and erratic rainfall. To address these challenges, this study leverages advanced meteorological data, XGBoost algorithm, and geospatial technologies to develop the NRWPS capable of providing real-time weather forecasts and early warning signals tailored for agricultural decision-making. By integrating satellite observations, local weather station data, and historical climate records, the system ensures high-resolution, reliable, and actionable weather information accessible to farmers, extension officers, and policymakers. This initiative aims to transform the region's adaptive capacity to climate change by minimizing climate-related risks, optimizing resource use. Furthermore, this study aligns with global and regional sustainability goals, offering a scalable solution for climate resilience in Sub-Saharan Africa while bolstering food security in the North Rift of Kenya and serving as a benchmark for similar interventions across the continent.

This research uniquely blends XGBoost and environmental data to strengthen climate mitigation measures as well as agricultural decision-making. The key contributions of the study are summarized in the following highlights outlining the crucial knowledge provided by this research.

• Integration of NDVI and BSI for Enhanced Predictive Accuracy: Leveraged XGBoost, as a means of improving the accuracy of NRWPS by first incorporating NDVI and BSI.
• Comprehensive Environmental Monitoring System: Created elaborate systems that integrate the NDVI, rainfall, temperature, and BSI to enhance the management of land and resources.
• Insights on Climate-Vegetation Interactions: Explained the role of temperature alterations after the year 2000 in the detriment of vegetation while showcasing the ability of rainfall to rejuvenate vegetation and help the equilibrium of the ecosystem.
• Detection of Land Degradation Trends: The increased BSI and decreased NDVI were demonstrated in the research as possible indicators for soil exposure due to decreased vegetation, revealing the expanding problem of land degradation in the North Rift of Kenya.
• Support for Sustainability and Resilience: Recommend NRWPS to be an important component for the viable strategies aimed at sustainability, food security, and climate change measures in the North Rift region.

The article is organized into the following sections: section 1, the Introduction, which establishes the study's context, objectives, and significance. Section 2 details the Materials and Methods and provides an overview of the methodology, including the study area and meteorology, datasets, and methods in 2.1, 2.2, 2.3 respectively. Additionally, section 3 details the Results of the study while section 4 provides a discussion of the results which highlights the key findings, including the model's predictive accuracy and its applications in monitoring vegetation and land degradation while contextualizing the findings within the existing literature, addressing their implications, strengths, and limitations. Finally, section 5 provides conclusions that summarize the study's contributions, emphasizing its potential for broader application and offering directions for future research and practical implementation.

## 2 Materials and Methods

### 2.1 Study area and meteorology

The North Rift region of Kenya, located between latitudes 1°N to 5°N and longitudes 34°E to 37°E (see Fig. 1 and Table 1), features a highly diverse climate shaped by its topography and atmospheric systems. Highland areas that include Uasin Gishu, Nandi, and Elgeyo-Marakwet counties experience temperate conditions, with average temperatures between 10 °C and 25 °C [1]. These counties receive annual rainfall ranging from 1000 to 1500 mm, mainly during the long rains (March-April-May (MAM)) and short rains (October-November-December (OND)), which is conducive for agriculture, particularly maize, wheat, and tea cultivation [10,11]. In contrast, low-lying counties such as Turkana, West Pokot, and Samburu exhibit a semi-arid to arid climate, with temperatures often exceeding 30 °C and lower, erratic rainfall amounts ranging from 200 to 600 mm annually. These conditions lead to recurrent droughts, making pastoralism a key livelihood in these areas [10,12]. The region's climate is largely influenced by climatic systems such as the Inter-Tropical Convergence Zone (ITCZ) and the Indian Ocean Dipole (IOD) [13,14]. These systems contribute to seasonal rainfall variability, which results in cycles of droughts and floods, affecting agricultural productivity and water resources in the region [11,15].

**Table 1:** The latitude and longitudes of the eight (8) counties represent Kenya's North-rift region Source; [1].

Name of the County	Latitude	Longitude	Population Source [39]:
Turkana	34° 30′ and 36° 40′ E	1° 30′ and 5° 30′ N	926,976
West Pokot	1° and 2° N	34° 47′ and 35° 49′ E	621,241
Elgeyo Marakwet	0° 20′ and 1° 30′ N	35° 0′ and 35° 45′ E	454,480
Baringo	0° 13′ S and 1° 40′ N	35° 36′ and 36° 30′ E	666,763
Nandi	0° 6′ 23.76″ N	35° 11′ 1.61″ E	885,711
Uasin Gishu	0° 3′ S and 0° 55′ N	34° 50′ E and 35° 37′ W	1,163,186
Trans Nzoia	1° 2′ 42″ N	34° 58′ 44″ E	990,341
Samburu	0° 30′ and 2° 45′ N	36° 15′ and 38° 10′ E	310,327

This region experiences a highly variable population distribution with higher densities noticed in the fertile highlands of Uasin Gishu and Trans Nzoia counties, where agriculture thrives due to favorable climatic conditions. In contrast, the semi-arid and arid counties like Turkana and Samburu have sparse populations, with pastoralism being the predominant livelihood, as the harsh climatic conditions limit agricultural activities (see Table 1).

### 2.2 Datasets

To perform trend analysis in the desired climatic variables, pretreatment procedures were applied to the monthly-derived datasets from Climate Hazards Group InfraRed Precipitation with Station data (CHIRPS) (precipitation) (from https://dwata.chc.ucsb.edu/products/CHIRPS-2.0/) [16] and TerraClimate (Minimum and Maximum Temperature) for over thirty years at five-year intervals from 1990 to 2022) over each of the eight (8) counties in the north rift region of Kenya [1]. Similarly, the Normalized Difference Vegetation Index (NDVI) values were obtained from Landsat images from the United States Geological Survey (USGS) website archives (https://earthexplorer.usgs.gov/). Images at a resolution of 30 m from the Landsat 4–5 Thematic Mapper (from 1995 to 2013) and Landsat 7 Enhanced Thematic Mapper plus (ETM+) (from 2014 to 2021) were utilized in the study. Soil variables are sourced from SoilGrids (https://soilgrids.org/), offering global predictions for properties among them the Bare Soil Index (BSI) utilized in the current study. The North Rift Weather Prediction System (NRWPS) leverages ArcGIS Pro 3.2 and the Colab notebook using Python programming to provide insight into the spatiotemporal variations and establish the interrelationships between the selected variables over the North Rift region. Table 2 details the information of all the datasets utilized in the study, including the product name, periods used, spatial and temporal resolutions, and link/reference to each dataset.

**Table 2:** Information on all the datasets utilized in the study.

Dataset/Product Name	Variable	Period Used	Spatial Resolution	Temporal Resolution	Link/Reference
Climate Hazards Group InfraRed Precipitation with Station Data (CHIRPS)	Precipitation	1995–2020	0.05° (∼5.5 km)	Monthly	https://dwata.chc.ucsb.edu/products/CHIRPS-2.0/ [10]
TerraClimate	Minimum and Maximum Temperature	1995–2020	4 km	Monthly	https://climate.northwestknowledge.net/TERRACLIMATE/
Landsat 4–5 Thematic Mapper	NDVI	1995–2013	30 m	16 day	https://earthexplorer.usgs.gov/
Landsat 7 Enhanced Thematic Mapper Plus (ETM+)	NDVI	2014–2020	30 m	16 day	https://earthexplorer.usgs.gov/
SoilGrids	BSI	1995–2020	250 m	Static	https://soilgrids.org/

To resolve inconsistencies in data temporal aggregation was used to harmonize the sampled datasets at varying intervals. Additionally, ArcGIS Pro 3.2 and Python functions (′regrid′, ′resample′, ′interpolate′) aided in realizing dataset alignment while bias correction was implemented to ensure consistency, thus improving trend analysis across climatic and environmental variables in the North Rift region of Kenya. Cloud masking was conducted using the Quality Assessment (QA) band from the Landsat datasets, utilizing the Fmask algorithm in ArcGIS Pro 3.2 and Google Earth Engine to filter out clouds and cloud shadows, which helps in providing an accurate representation of NDVI.

### 2.3 Methods

#### 2.3.1 Extreme gradient boosting (XGBoost)

XGBoost is a high-performance machine learning algorithm designed for structured data, leveraging gradient boosting principles to sequentially minimize errors [17]. Its optimization objective combines a loss function $L\left(y_{i},{ŷ}_{i}\right)$ with a regularization term, $\Omega \left(f_{k}\right)$, to balance model accuracy and complexity, reducing overfitting. The model predicts outcomes by summing the outputs of multiple decision trees:

$${ŷ}_{i}=\sum_{k=1}^{K}f_{k}\left(x_{i}\right),f_{k}\in Ƒ$$

Here, $\left(f_{k}\right)$, represents individual regression trees. Key features include advanced regularization (L1 and L2), efficient handling of missing data, and scalability through parallel and distributed computing [18]. While requiring careful hyperparameter tuning, XGBoost excels in modeling complex, non-linear relationships and handling large-scale, sparse datasets, making it a cornerstone in predictive modeling across diverse applications [19].

#### 2.3.2 North rift weather prediction system (NRWPS) model development

**Fig. 2:**
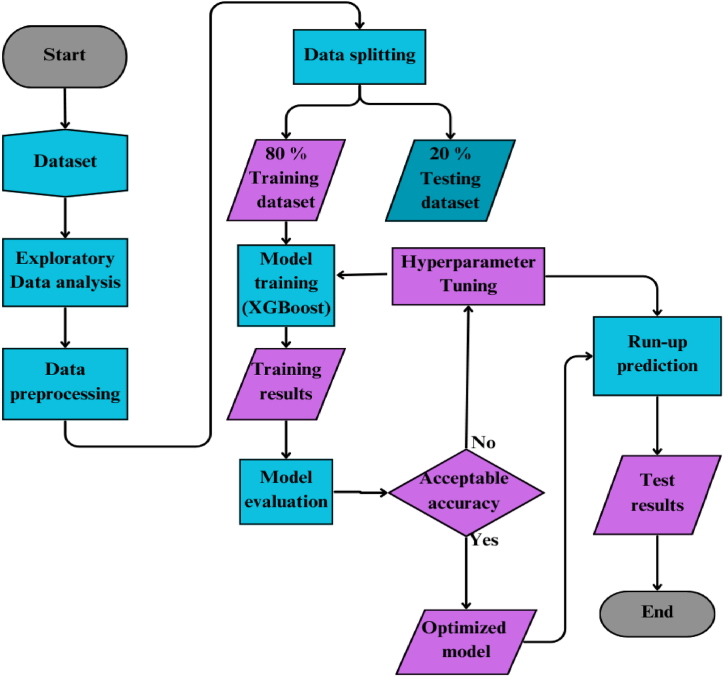
Flowchart for developing the North Rift Weather Prediction System, from data cleaning to model training and integration.

The North Rift Weather Prediction System (NRWPS) model was developed using a systematic methodology outline in Fig. 2 which integrates data cleaning, feature engineering, normalization, and XGBoost algorithm to enhance predictive accuracy based on environmental and climatic interactions. A flow chart summary of the implementation process of the NRWPS is highlighted in Fig. 2.

This process starts with data loading and exploratory cleaning, where environmental indices i.e. NDVI and BSI, precipitation, and temperature data (1995–2020) are imported into a DataFrame (merged_df) and cleaned. Feature engineering follows, involving the creation of interaction-based features such as averages and anomalies by combining precipitation, minimum, and maximum temperatures and conducting statistical analyses to extract trends. The dataset is then split into training (80 %) and testing (20 %) sets using train_test_split, and features are normalized with StandardScaler to ensure consistent scaling. The XGBoost algorithm is employed for model training using parameters such as squared error loss, root mean squared error metric, a learning rate of 0.1, maximum depth of 6, and 100 boosting rounds. The trained model is evaluated on the testing set with performance metrics including Mean Absolute Error (MAE), Mean Squared Error (MSE), and R-squared (R^2^) as discussed in the proceeding sections. Finally, the NRWPS model is delivered, incorporating insights from precipitation, temperature, and vegetation interactions.

#### 2.3.3 Data loading and exploratory data cleaning

The first step in building the BSI, and NDVI models involved loading the relevant data into the working environment. The dataset, which included various environmental and vegetation indices from 1995 to 2020, was imported into a pandas Data Frame called merged_df. After loading the data, exploratory data cleaning was performed to ensure the quality and integrity of the dataset.

#### 2.3.4 Data splitting and feature normalization

Once the data was cleaned and preprocessed, it was split into training and testing sets using the train_test_split function from the sklearn.model_selection module. The test set size was set to 20 % of the total data, and a random state of forty-two (42) was used for reproducibility. This split allowed for the evaluation of the model's performance on unseen data and helped prevent overfitting. Before training the models, feature normalization was applied to ensure that all the features were on a similar scale. The StandardScaler from sklearn.preprocessing was used to standardize the features by subtracting the mean and scaling to unit variance. The scaler was fit on the training data and then applied to both the training and testing sets to avoid information leakage. Feature normalization helps improve the convergence speed and stability of the learning algorithms, especially when dealing with features that have different scales or units.

#### 2.3.5 Model training and evaluation

The BSI, and NDVI models were built using the XGBoost algorithm, an optimized gradient-boosting framework known for its excellent performance and speed. The XGBoost model was set up with the following parameters: regression with squared error loss, Evaluation Metric that involves the root mean squared error, Learning Rate (eta): 0.1, and Maximum Depth: 6.

The models were trained using the xgb.train function with the specified parameters and the training data. The number of boosting rounds was set to 100. After training, the models were evaluated on the testing set using the predict function. Several evaluation metrics were calculated to assess the performance of the models.

● MAE: Measures the average absolute difference between the predicted and actual values [20].

$$\text{MAE}=\frac{1}{n}\sum_{i=1}^{n}\left|y_{i}-{ŷ}_{i}\right|$$

Where n is the number of observations, $y_{i}$ is the actual value and ${ŷ}_{i}$ is the predicted value.

● MSE: Measures the average squared difference between the predicted and actual values [21].

$$\text{MSE}=\frac{1}{n}\sum_{i=1}^{n}{\left(y_{i}-{ŷ}_{i}\right)}^{2}$$

Where n is the number of observations, $y_{i}$ is the actual value and ${ŷ}_{i}$ is the predicted value.

● R^2^ score: Represents the proportion of variance in the target variable that is predictable from the features [22].

$$R^{2}=1-\frac{\sum_{i=1}^{n}{\left(y_{i}-{ŷ}_{i}\right)}^{2}}{\sum_{i=1}^{n}{\left(y_{i}-{ȳ}_{i}\right)}^{2}}$$

Where n is the number of observations, $y_{i}$ is the actual value, ${ŷ}_{i}$ is the predicted value, ${ȳ}_{i}$ is the mean of the actual values while $\sum_{i=1}^{n}{\left(y_{i}-{ȳ}_{i}\right)}^{2}$ is the total sum of squares. These metrics were used to assess the performance of the NRWPS model by comparing actual values to the model's predictions.

## 3 Results

### 3.1 Annual trends

Table 3 provides a synopsis of key annual trends from NDVI, precipitation, minimum and maximum temperature as well as the Bare Soil Index (BSI) for the period 1995 to 2020, with a margin of error of ±5 % for all the parameters.

**Table 3:** Annual trend analysis data for NDVI, precipitation, temperature, and BSI for the years 1995, 2000, 2005,2011, 2016, 2020.

Year	Annual NDVI	Annual Precipitation	Minimum Temperature	Maximum Temperature	BSI
1995	0.19 ± 0.0095	457 ± 23	17.6 ± 0.88	32 ± 1.6	0.0376 ± 0.0019
2000	0.171 ± 0.0085	365 ± 18	17.9 ± 0.89	32.3 ± 1.6	0.0778 ± 0.0039
2005	0.208 ± 0.01	496 ± 25	17.6 ± 0.88	32.6 ± 1.6	0.0404 ± 0.002
2011	0.228 ± 0.011	663 ± 33	18.2 ± 0.91	32.5 ± 1.6	−0.00159 ± −7.9e-05
2016	0.281 ± 0.014	492 ± 25	18.3 ± 0.91	32.5 ± 1.6	0.000863 ± 4.3e-05
2020	0.373 ± 0.019	854 ± 43	18.5 ± 0.93	31.7 ± 1.6	−0.0551 ± −0.0028

**Fig. 3:**
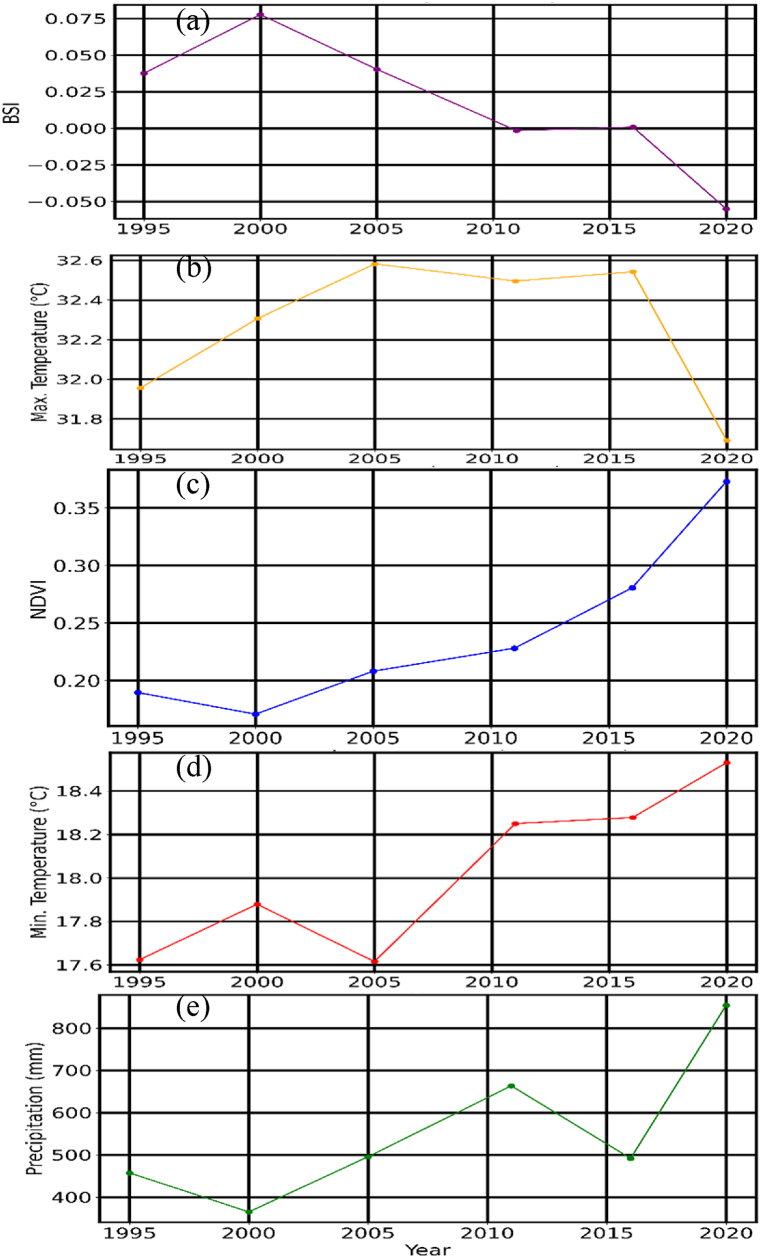
Temporal trends of environmental and climatic variables from 1995 to 2020. (a) Bare Soil Index (BSI). (b) Maximum temperature (°C). (c) Normalized Difference Vegetation Index (NDVI). (d) Minimum temperature (°C). (e) Annual precipitation (mm).

Similarly, Fig. 3 shows the trends over the same period and the key to note is the relationships between Bare Soil Index (BSI) (Fig. 3(a)), maximum temperature (Fig. 3(b)), NDVI (Fig. 3(c)), minimum temperature (Fig. 3(d)), and precipitation (Fig. 3(e)).

### 3.2 NRWPS model prediction

The NRWPS was utilized as a prediction model to provide localized and accurate weather information for the North Rift region of Kenya. Table 4 presents the predicted NDVI and BSI values for various coordinate points, from historical data spanning from 1995 to 2020.

**Table 4:** Predicted NDVI and BSI values for various coordinate points, along with historical precipitation data spanning from 1995 to 2020.

Point ID	Precipitation, BSI, NDVI, Maximum and Minimum temperature from 1995 to 2020	BSI 2023	NDVI 2023	Predicted BSI	Predicted NDVI
1791	See supplementary data provided	0.031879	0.325345	0.040051	0.310291
32		0.097281	0.284436	0.168509	0.285657
2031		0.117978	0.120877	0.11728	0.123042
4688		−0.339014	0.77622	−0.446288	0.756530
1725		−0.027105	0.294312	−0.124191	0.265693
335		−0.173453	0.519890	−0.379294	0.529295
845		0.042381	0.156929	0.089309	0.156941
3001		0.042687	0.72105	0.067441	0.86388

### 3.3 Spatial-temporal characterization and interactions between selected parameters

**Fig. 4:**
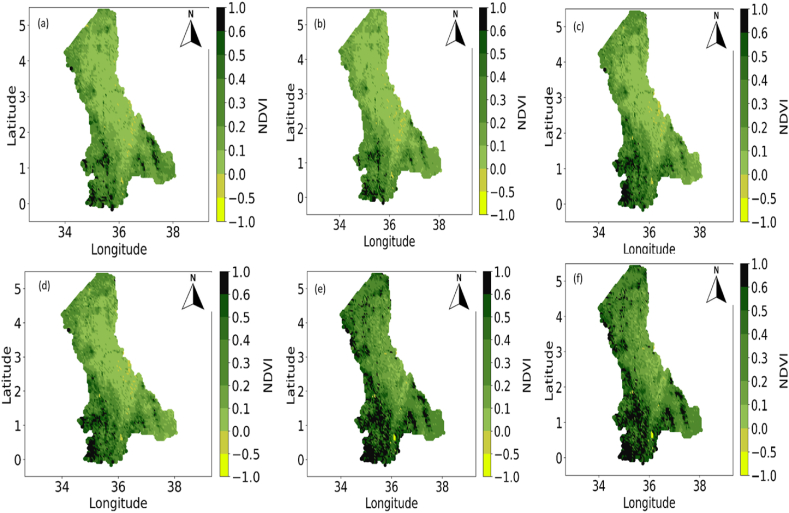
Spatial variations of Normalized Difference Vegetation Index (NDVI) for (a) 1995, (b) 2000, (c) 2005, (d) 2011, (e) 2016, and (f) 2020 over the north rift counties.

**Fig. 5:**
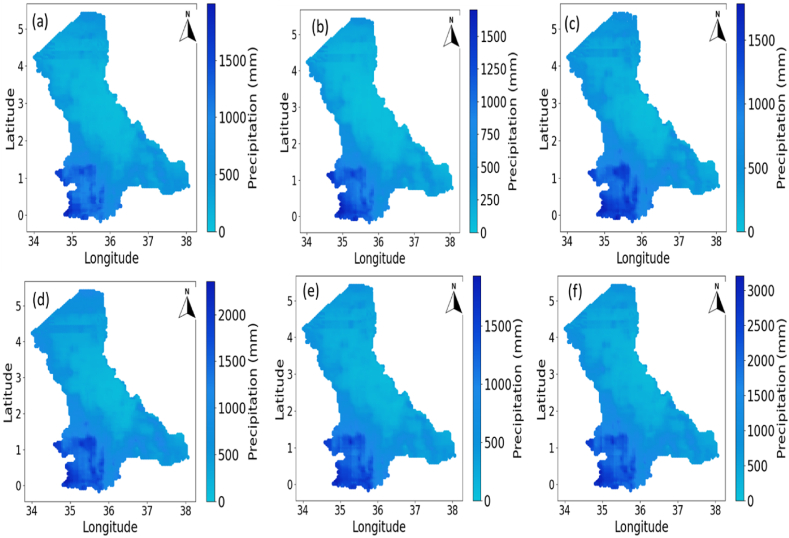
Spatial variations of Precipitation for (a) 1995, (b) 2000, (c) 2005, (d) 2011, (e) 2016, and (f) 2020 over the North Rift counties.

**Fig. 6:**
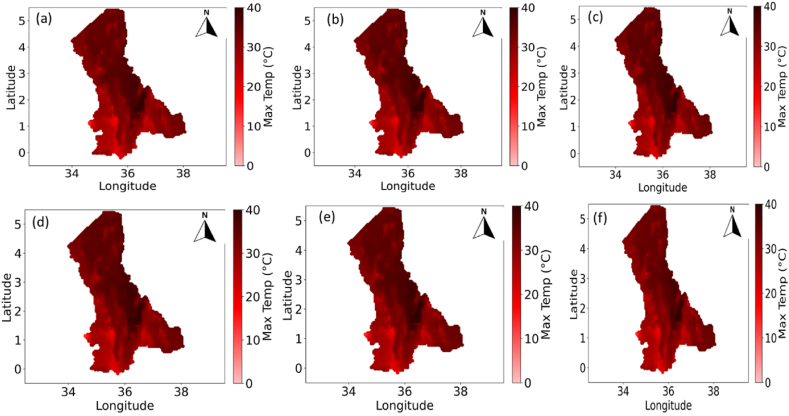
Spatial variations of Maximum Temperature for (a) 1995, (b) 2000, (c) 2005, (d) 2011, (e) 2016, and (f) 2020 over the North Rift counties.

**Fig. 7:**
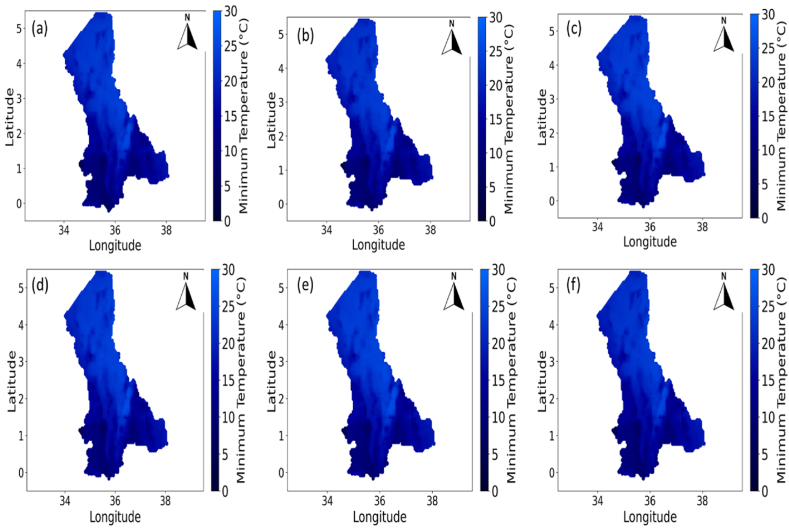
Spatial variations of Minimum Temperature for (a) 1995, (b) 2000, (c) 2005, (d) 2011, (e) 2016, and (f) 2020 over the North Rift counties.

**Fig. 8:**
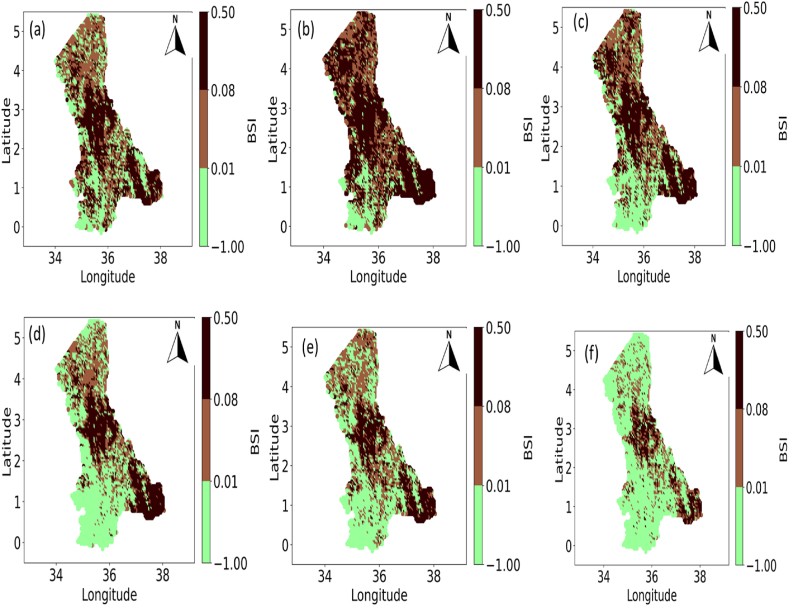
Spatial variations of Bare Soil Index (BSI) for (a) 1995, (b) 2000, (c) 2005, (d) 2011, (e) 2016, and (f) 2020 over the North Rift counties.

The NRWPS was also used to analyze the spatiotemporal characteristics in NDVI (in Fig. 4), Precipitation (in Fig. 5), Maximum (in Fig. 6), and Minimum (in Fig. 7) Temperatures, and BSI (in Fig. 8) to understand their interrelationships from 1995 to 2020 over the North Rift region of Kenya.

## 4 Discussions

### 4.1 Annual trends in selected parameters

Table 3 and Fig. 3 highlight temporal variations in the Bare Soil Index (BSI) (see Fig. 3(a)), maximum temperature (Fig. 3(b)), NDVI (Fig. 3(c)), minimum temperature (Fig. 3(d)), and precipitation (Fig. 3(e)) between 1995 and 2020. The BSI trend in Fig. 3(a) indicates an initial increase followed by a sharp decline after 2015 implying a reduction in bare soil areas in the North Rift region. This may be associated with land cover changes such as reforestation efforts or shifts in agricultural practices, consistent with global conservation initiatives reported by Ref. [23], or the influence of precipitation (see Fig. 3(e)) that is observed to be increasing over the region at the same time. Both maximum (see Fig. 3(b)) and minimum (see Fig. 3(d)) temperatures show an increasing trend, with maximum temperatures peaking around 2005–2015 before a slight decline in 2020, while minimum temperatures exhibit steady growth post-2005. Indeed, there is evidence of declining diurnal temperature over the region as earlier reported by Ref. [1]. These trends align with global warming patterns selected meteorological parameters observed across Africa, as highlighted in the IPCC Sixth Assessment Report [24].

NDVI (see Fig. 3(c)) is indicative of a significant upward trend, confirming improved vegetation health and density, which could be linked to increased rainfall [1] and the overall decline in BSI (see Fig. 3(a)) over the region. On the other hand, precipitation shows marked variability, with peaks around 2015 and 2020 that are a characteristic influence of large-scale climate phenomena like El Niño over the region [1,25]. Overall, the observed trends (see Fig. 3) suggest a dynamic interplay between climatic factors and land cover dynamics, with the increasing NDVI (in Fig. 3(c)) and declining BSI (in Fig. 3(a)) indicating potential benefits from conservation and land use changes over the North Rift region of Kenya.

### 4.2 NRWPS prediction results

To understand the NRWPS prediction results, we refer to Table 4 where NDVI and BSI values for various coordinate points within the region, along with historical precipitation data spanning from 1995 to 2020 have been utilized. In the present study, we have leveraged the NRWPS to predict BSI and NDVI values which are crucial for ecosystem monitoring including vegetation health and soil exposure. The evaluation of the BSI and NDVI models yielded promising results, indicating their effectiveness in predicting these indices (see Table 4). The NRWPS for BSI achieved an MSE of 0.029, an MAE of 0.019, and an R-squared score of 0.93. These metrics demonstrate the model's ability to accurately predict BSI values, with relatively low error values and a high proportion of explained variance [20]. Similarly, the prediction of NDVI values exhibited strong performance, with an MAE of 0.024, an MSE of 0.002, and an R-squared score of 0.945 [20]. The low error values and high R-squared score in predicting both BSI and NDVI suggest that the NRWPS can effectively capture the relationships between environmental factors and vegetation health, providing accurate predictions.

At Point 3001 (36.457915, 1.678547), the predicted NDVI value for 2023 is 0.106, while the predicted BSI value is 0.043. The precipitation values at this point show an increasing trend from 1995 to 2016, followed by a slight decrease in 2020. Moving to Point 845 (35.702001, 4.144390), the predicted NDVI value for 2023 is 0.284, and the predicted BSI value is 0.042. The precipitation values at this location exhibit an overall increasing trend from 1995 to 2016, with a decline observed in 2020. At Point 335 (35.257346, 4.413391), the predicted NDVI value for 2023 is 0.701, and the predicted BSI value is −0.173. The precipitation values at this point show a consistent increase from 1995 to 2016, followed by a decrease in 2020.

Similarly, at Point 1725 (34.946088, 3.023552), the predicted NDVI value for 2023 is 0.409, and the predicted BSI value is −0.027. The precipitation values at this location display an increasing trend from 1995 to 2016, with a slight decline in 2020. Finally, at Point 4688 (34.990553, 0.333542), the predicted NDVI value for 2023 is 0.779, and the predicted BSI value is −0.339. The precipitation values at this point exhibit a steady increase from 1995 to 2016, with a notable decrease in 2020. These observations highlight the variations in predicted NDVI and BSI values across different coordinate points and the corresponding trends in historical precipitation data. The increasing trends in precipitation from 1995 to 2016, followed by decreases in 2020, provide insights into the potential factors influencing the predicted vegetation health and soil exposure at these locations.

The successful development of the NRWPS opens up various applications and opportunities for understanding and managing ecosystems including vegetation health. By accurately predicting BSI and NDVI values, stakeholders can gain valuable insights into vegetation dynamics, soil exposure, and the impacts of environmental factors on ecosystem health. The insights from the NRWPS can inform land management strategies, conservation efforts, and agricultural practices, enabling data-driven decision-making and promoting sustainable resource utilization. Moreover, NRWPS can be integrated into larger decision support systems or early warning mechanisms, allowing for proactive interventions and targeted actions for overall livelihood improvement.

### 4.3 Model evaluation

The BSI prediction model demonstrates strong performance, as evidenced by its low MSE value of 0.029 and MAE value of 0.0189. These metrics indicate that the NRWPS is closely aligned with the observed BSI values, with relatively small average differences between the predicted and actual values. The low error metrics suggest that NRWPS can effectively capture the underlying patterns and relationships in the data. Moreover, the BSI model's R-squared score of 0.933 is remarkably high, indicating a strong correlation between the predicted and actual BSI values. This score implies that the model explains approximately 93.26 % of the variance in the data, further confirming its ability to accurately represent the observed bare soil exposure patterns [22].

Similarly, the NRWPS prediction of NDVI exhibits exceptional performance i.e. MSE value of 0.002 and MAE value of 0.024 highlight its ability to generate highly accurate predictions of vegetation health. The low MSE value indicates minimal average squared differences between the predicted and actual NDVI values, while the low MAE value suggests that the model's predictions closely match the observed vegetation conditions. Additionally, the R-squared score of 0.945 for NDVI is exceptionally high, showcasing its strong predictive power [22]. The comparative evaluation of the BSI and NDVI models reveals that both models perform exceptionally well, with the NDVI model slightly outperforming the BSI model in terms of predictive accuracy. This superior performance can be attributed to the XGBoost algorithm's ability to effectively learn from the training data and make precise predictions based on the selected features and model parameters [17].

### 4.4 Spatial-temporal characterization

The spatial and temporal characteristics observed across the figures reveal critical insights into environmental trends in the North Rift counties. Specifically, Fig. 4, illustrates NDVI over six time periods (in years) i.e. 1995, 2000, 2005, 2011, 2016, and 2020. Spatial variability in vegetation health (herein NDVI), is significantly high with denser vegetation concentrated in high-altitude areas of Trans Nzoia, Uasin Gishu, and Nandi counties and lower values in arid and semi-arid zones of Turkana, West Pokot, Elgeyo-Marakwet, Baringo, and Samburu counties (see Table 1). Temporal analysis reveals fluctuations in NDVI that are attributed to climatic factors like droughts and rainfall variability [1]. Observed declines in NDVI may suggest vegetation degradation, whereas periods of improvement reflect recovery driven by reforestation, land management practices, or favorable climatic conditions. Specifically, in 1995 (Fig. 4(a)), NDVI visualization indicated relatively healthy vegetation, particularly in the northern parts of the North Rift region i.e. arid and semi-arid areas, as reflected by the predominance of green shades, representing higher NDVI values. However, over time, a decline in NDVI is observed, particularly around 2005 (Fig. 4(c) and beyond. By 2016 and 2020, this decline became more pronounced, indicating that vegetation health had deteriorated across several parts of the region. The reduction in NDVI, especially in the southern and central areas of the region, suggests a progressive degradation of vegetation cover, which can be attributed to several factors, including rising temperatures, deforestation, and changing land-use practices [26,27].

In Fig. 5, precipitation patterns exhibit significant spatial heterogeneity across the North Rift counties, mirroring the spatial variability seen in NDVI. Areas receiving higher rainfall generally correspond to regions with higher NDVI values as seen in Fig. 4, highlighting the direct dependence of vegetation health on precipitation [28]. Temporally, the precipitation data reflects inter-annual variability, with some years exhibiting drought conditions, such as 2005 (see Fig. 5 (b)), which aligned with low NDVI values as seen in Fig. 4 (b) [1]. The spatial distribution also shows a possible orographic influence, with higher rainfall recorded in regions near elevated terrain. These trends underscore the critical role of precipitation as a driver of vegetation dynamics and land productivity [29], an important observation given that the entire region relies on rain-fed agriculture.

Fig. 6, Fig. 7, which present maximum and minimum temperature variations respectively, further explain patterns in vegetation and soil characteristics. In Fig. 6, areas with consistently high maximum temperatures across the study period from 1995 (Fig. 6(a)), 2000 (Fig. 6(b)), 2005 (Fig. 6(c)), 2011 (Fig. 6(d)), 2016 (Fig. 6(e)), and 2020 (Fig. 6(f)) align with regions of reduced vegetation cover in Fig. 4. This highlights extreme heat's stress on vegetation, especially in water-limited environments [28]. Meanwhile, Fig. 7 demonstrates how minimum temperature trends, particularly warming nights, can exacerbate stress on ecosystems by reducing diurnal cooling, essential for plant recovery. Temporal trends in both figures reveal increasing temperatures, consistent with global warming trends, and their spatial interplay with other climatic variables reinforces the need for integrated management of climate impacts [[29], [30], [31]]. In 1995, minimum temperatures (see Fig. 7 (a)) were widespread, especially in the northern regions. These cooler conditions are important for plant respiration, as they allow vegetation to conserve energy during the night. As the years progressed, a consistent warming trend in minimum temperatures was observed, particularly evident from 2000 (Fig. 7(b)), 2005 (Fig. 7(c)), 2016 (Fig. 7(e)), and 2020 (Fig. 7(f)), when minimum temperatures were significantly higher therefore increasing night-time vegetation respiration rates. The relationship between warmer minimum temperatures and declining NDVI suggests that vegetation is increasingly stressed due to the lack of cooler night-time conditions [29].

The bare soil index (BSI) depicted in Fig. 8 complements the analysis of vegetation and climatic parameters. High BSI values align with areas of low NDVI in Fig. 4 and low precipitation in Fig. 5, reflecting regions where vegetation has been replaced by exposed soil due to degradation or aridity. The temporal trends in Fig. 8 indicate that bare soil exposure has increased over time in certain areas, possibly due to deforestation, overgrazing, and agricultural expansion. Conversely, areas with reduced BSI suggest efforts toward land restoration or natural vegetation recovery. The spatial alignment of high BSI with regions experiencing high temperatures in Fig. 6, Fig. 7 further underscores the compounded effects of thermal stress and insufficient precipitation on land degradation [29].

### 4.5 Interactions in selected parameters

#### 4.5.1 Interactions between NDVI and precipitation

The interplay between NDVI in Fig. 4 and precipitation in Fig. 5 illustrates a direct relationship: regions with higher rainfall often exhibit higher NDVI values, emphasizing precipitation as a primary driver of vegetation health. Periods of low NDVI, such as in 2005, correspond to years with reduced rainfall, indicating drought-induced stress [30]. In 1995, the NDVI (in Fig. 4 (a)) showed relatively healthy vegetation across much of the North Rift region, particularly in the northern and central parts, where the green shades dominate, reflecting higher NDVI values. These areas correlate with regions of moderate to high precipitation, as shown in the precipitation (in Fig. 5 (a)) for the same year. Rainfall levels exceed 1000 mm in certain areas, providing the necessary moisture for plants to grow and maintain healthy biomass. The balance between adequate rainfall and vegetation health is established during this period, as the water availability allows for dense vegetation cover, resulting in higher NDVI values [32].

By 2000, the NDVI patterns started to shift (see Figure 4(b)), especially in the southern regions, where a decline in vegetation density was noticeable. The yellowish hues in Figure 4(b) reflect lower vegetation density, indicating stress or reduced growth. The precipitation in Fig. 5(b) shows a reduction in rainfall in these areas, with lighter blue shades indicating less precipitation compared to that in Fig. 4(a) in 1995. This decrease in precipitation directly impacts the vegetation, as lower rainfall limits water availability for plants, reducing their ability to photosynthesize and grow. As a result, NDVI values drop, demonstrating the strong dependence of vegetation on consistent and adequate rainfall [29].

Fig. 4(c) shows a declining trend in NDVI in 2005 over most parts of the North Rift region, particularly in areas that experienced further reductions in rainfall. The precipitation as seen in Fig. 5 (c) shows large sections of the region received less than 1000 mm of rainfall, particularly in the central and northern areas. This decrease in rainfall corresponds to lower NDVI values (see Fig. 4(c), as vegetation in these areas becomes more stressed due to water scarcity. The relationship between precipitation and NDVI becomes increasingly evident: where precipitation is lower, NDVI values drop, illustrating how crucial water availability is for maintaining healthy vegetation cover. Reduced rainfall leads to water stress in plants, limiting their growth and causing a decline in vegetation density [33].

In 2011, there was some recovery in NDVI values (see Fig. 4 (d)) in areas that previously showed signs of vegetation stress. This recovery is most prominent in regions where the precipitation (see Fig. 5 (d)) indicates an increase in rainfall. The relationship between precipitation and vegetation health remains clear: higher rainfall supports vegetation growth, leading to higher NDVI values, while water scarcity results in reduced vegetation cover [31]. The NDVI patterns in 2016 (see Fig. 4 (e)) exhibit more variability across the region, with some areas showing higher vegetation density, while others continue to experience stress. This variability is directly tied to the uneven distribution of rainfall, as shown in the precipitation (see Fig. 5 (e)) for 2016 and similarly for 2020 (see Fig. 5 (f)).

Overall, the interaction between NDVI and precipitation from 1995 to 2020 highlights the direct influence of rainfall on vegetation health. It is demonstrated that precipitation serves as the key factor that supports plant growth and maintains vegetation cover. When rainfall is abundant, NDVI values are high, reflecting healthy vegetation, while periods of reduced rainfall lead to declines in NDVI, indicating stressed or sparse vegetation. This relationship emphasizes the significance of understanding precipitation patterns in the context of ecosystem management, climate adaptation, and agricultural productivity, as changes in rainfall directly impact the capacity of ecosystems to support biodiversity and maintain ecosystem services [29].

#### 4.5.2 Interactions between NDVI and maximum and minimum temperature

The relationship between NDVI and temperatures in Fig. 6, Fig. 7 highlights how high maximum and minimum temperatures respectively exacerbate vegetation stress, particularly in areas already experiencing water deficits [29]. In 1995, temperature in Fig. 6, Fig. 7) were lower, especially in the northern regions, as indicated by the lighter red hues. However, by 2011 and beyond, these temperatures increase significantly (see Fig. 6, Fig. 7), with the red coloration becoming more intense and widespread. The rise in maximum temperatures is of particular importance for vegetation, as high maximum temperatures increase evapotranspiration rates, leading to faster drying of soils. This, in turn, reduces the moisture available for plants, exacerbating water stress and limiting their ability to grow and thrive [30]. The increase in maximum temperatures coincides with the decline in NDVI (see Fig. 4, Fig. 6 across all years), indicating that vegetation is struggling to cope with the increasing heat stress [31]. The relationship between NDVI and maximum temperature is evident across the time series and as the climate becomes warmer, the ability of vegetation to recover from heat and water stress diminishes, resulting in the observed decline in NDVI [34].

Consistent rise in temperatures, both maximum and minimum as seen in Fig. 6, Fig. 7 (across all years of study) respectively, is a key driver of the observed changes in vegetation health (as observed in the NDVI values (in Fig. 4 across all years). Warmer conditions lead to reduced water availability, higher evapotranspiration rates, and increased plant respiration, all of which contribute to declining NDVI. As temperatures continue to rise, these challenges are likely to intensify, with further reductions in vegetation density, increased desertification, and greater risks to food security in the region [35]. Additionally, increasing temperatures (both minimum and maximum) have a clear negative impact on NDVI, suggesting that vegetation is increasingly stressed by warmer conditions, therefore underscoring the urgent need for climate adaptation strategies to mitigate the impacts of rising temperatures on ecosystems and agricultural systems in this region.

#### 4.5.3 Interactions between NDVI and bare soil index (BSI)

Interactions between NDVI (in Fig. 4) and BSI (in Fig. 8) for the years under study show that regions with lower NDVI often correspond to higher BSI values. This is a pointer towards land degradation and loss of vegetative cover in the region as earlier confirmed by Ref. [29]. In 1995, NDVI visualization (see Fig. 4 (a)) revealed widespread healthy vegetation across most of the region, especially in the northern and central parts, with relatively high NDVI values (green shades). Simultaneously, the BSI (see Fig. 8 (a)) shows lower values of BSI exposure in these regions. The presence of dense vegetation inhibits soil exposure, contributing to lower BSI values. In areas where BSI is higher, the NDVI values are lower, indicating less vegetation cover and higher soil exposure, likely due to land degradation or naturally sparse vegetation [32].

By 2000, a noticeable shift occurred in both the NDVI (see Fig. 4 (b) and BSI (see Fig. 8 (b)) where the latter shows a decrease in vegetation density, particularly in the southern regions. Correspondingly, Fig. 8 (b) shows an increase in bare soil exposure in these areas. The inverse relationship between NDVI and BSI became more pronounced in 2000, illustrating how an increase in bare soil directly impacts vegetation health by limiting the available soil moisture, increasing susceptibility to erosion, and reducing plant growth [28,32,36]. In 2005, the trend continued with further reductions in NDVI (see Fig. 4 (c)) particularly in areas with significant increases in bare soil exposure, as reflected in the BSI (see Fig. 8 (c)). In 2011, Fig. 4 (d) for the NDVI shows some recovery while Fig. 8 (d) for BSI demonstrates a reduction, particularly in the central and northern regions. However, in other regions, especially in the southern parts, NDVI (see Fig. 4 (d)) remains low, and BSI (see Fig. 8 (d)) remains high, indicating persistent land degradation and poor vegetation recovery.

In 2016, the NDVI (see Fig. 4 (e)) and BSI (see Fig. 8 (e)) showed a more mixed pattern. In some regions, NDVI improves, reflecting higher vegetation density, while in others, particularly in the southern and eastern areas, NDVI remains low, and BSI remains high. This indicates that bare soil exposure continues to limit vegetation growth in these areas which points to the fact that vegetation struggles to recover in these areas, leading to persistently low NDVI values [37,38]. Likewise, in 2020, the relationship between NDVI (see Fig. 4 (f)) and BSI (see Fig. 8 (f)) remains evident. In summary, the temporal progression of NDVI and BSI from 1995 to 2020 highlights the inverse relationship between vegetation health and bare soil exposure. As bare soil exposure increases (as seen in higher BSI values), vegetation density declines (reflected in lower NDVI values), and vice versa. This relationship is a critical indicator of land degradation processes such as desertification, deforestation, and unsustainable land use practices, which expose soil to the elements, leading to erosion, nutrient loss, and a reduction in the land's ability to support vegetation.

#### 4.5.4 Interactions between precipitation and bare soil index (BSI)

The relationship between BSI in Fig. 8 and precipitation in Fig. 5 across all the years of study is evident, with higher bare soil index values in regions of low rainfall. This spatial correlation highlights how insufficient precipitation leads to reduced vegetation cover and increased soil exposure. Additionally, the BSI (see Fig. 8) interaction with Maximum temperature (Fig. 6) and Minimum temperature (Fig. 7) shows that regions with high temperatures are more likely to experience increased bare soil exposure, as high heat accelerates soil moisture loss and inhibits vegetation growth [37] across the years of study. These interactions emphasize the compounded impacts of low precipitation and high temperatures on land degradation, as supported by Ref. [38]. Temporal trends in BSI also reflect climatic variability and land use changes, further reinforcing the need for integrated climate and land management strategies.

## 5 Conclusion

The integration of NDVI and BSI predictions using the XGBoost algorithm has significantly enhanced the North Rift Weather Prediction System (NRWPS), offering an advanced tool for agricultural and land management practices. The high predictive accuracy of the NRWPS provides reliable insights into vegetation health and soil exposure, facilitating better decision-making in crop management, irrigation planning, and land degradation monitoring. By combining weather forecasting with NDVI and BSI data, NRWPS serves as a comprehensive environmental monitoring system, playing a crucial role in supporting sustainable agriculture, soil conservation, and climate adaptation in the North Rift region of Kenya. The combined analysis of NDVI, BSI, temperature, and precipitation from 1995 to 2020 reveals key trends in vegetation health and land degradation. The observed decline in NDVI in regions with increasing BSI values highlights the adverse impact of land degradation and soil exposure on vegetation density. Climatic factors, particularly higher maximum temperatures after 2000, exacerbate vegetation loss, while regions with higher precipitation demonstrate healthier vegetation, emphasizing the importance of water availability for sustaining plant growth. This study underscores the critical need for sustainable land management practices to reduce soil exposure, combat deforestation, and mitigate the impacts of climate change. The successful application of the NRWPS showcases the potential for broader community impact, supporting food security, climate resilience, and environmental sustainability in the North Rift region. In conclusion, integrating NDVI and BSI models within decision support systems offers a promising approach to addressing the challenges posed by land degradation and climate change in vulnerable ecosystems such as the North Rift region of Kenya.

## CRediT authorship contribution statement

**John W. Makokha:** Writing – review & editing, Writing – original draft, Supervision, Project administration, Methodology, Investigation, Formal analysis, Conceptualization. **Peter W. Barasa:** Visualization, Validation, Formal analysis, Conceptualization. **Geoffrey W. Khamala:** Data curation.

## Compliance with ethical standards

None to declare.

## Data availability statement

Data will be made available on request. For requesting data, please write to the corresponding author.

## Additional information

The NRWPS is hosted on: https://public.tableau.com/app/profile/melisa.michuki/viz/KibabiiAnalysis_17121587548620/Tempcomp2011.

## Funding statement

This work is supported by a Research Grant (109705-001/002) from the Responsible Artificial Intelligence Network for Climate Action in Africa (RAINCA).

## Declaration of competing interest

The authors declare the following financial interests/personal relationships which may be considered as potential competing interests: John W. Makokha reports administrative support was provided by Kibabii University. John W. Makokha reports financial support was provided by Responsible Artificial Intelligence Network for Climate Action in Africa (RAINCA) consortium. If there are other authors, they declare that they have no known competing financial interests or personal relationships that could have appeared to influence the work reported in this paper.
